# Technological and Nutritional Aspects of Bread Production: An Overview of Current Status and Future Challenges

**DOI:** 10.3390/foods13132062

**Published:** 2024-06-28

**Authors:** Mariana Mesta-Corral, Ricardo Gómez-García, Nagamani Balagurusamy, Cristian Torres-León, Ayerim Y. Hernández-Almanza

**Affiliations:** 1 Food Products Research and Development Lab, Unidad Torreón, School of Biological Science, Universidad Autonoma de Coahuila, Torreón 27276, Coahuila, Mexico; mestam@uadec.edu.mx; 2 Center for Interdisciplinary Studies and Research, Unidad Saltillo, Universidad Autonoma de Coahuila, Arteaga 25350, Coahuila, Mexico; 3 CBQF—Centro de Biotecnologia e Química Fina—Laboratório Associado, Escola Superior de Biotecnologia, Universidade Católica Portuguesa, Rua Diogo Botelho 1327, 4169-005 Porto, Portugal; rgarcia@ucp.pt; 4 Laboratorio de Biorremediación, Unidad Torreón, Facultad de Ciencias Biológicas, Universidad Autonoma de Coahuila, Torreón 27276, Coahuila, Mexico; bnagamani@uadec.edu.mx; 5 Research Center and Ethnobiological Garden, Unidad Torreón, Universidad Autonoma de Coahuila, Viesca 27480, Coahuila, Mexico; 6 Agri-Food and Agro-Industrial Bioeconomy Research Group, Unidad Torreón, Universidad Autonoma de Coahuila, Torreón 27276, Coahuila, Mexico

**Keywords:** wheat bread, dietary fiber, organoleptic characteristics, enhanced food, health benefits, leguminous flours

## Abstract

Bakery products, especially bread, exist in many homes worldwide. One of the main reasons for its high consumption is that the main raw material is wheat, a cereal that can adapt to a wide variety of soils and climates. However, the nutritional quality of this raw material decreases during its industrial processing, decreasing the value of fibers, proteins, and minerals. Therefore, bread has become a product of high interest to increase its nutritional value. Due to the high consumption of bread, this paper provides a general description of the physicochemical and rheological changes of the dough, as well as the sensory properties of bread by incorporating alternative flours such as beans, lentils, and soy (among others). The reviewed data show that alternative flours can improve fiber, macro, and micronutrient content. The high fiber content reduces the quality of the texture of the products. However, new processing steps or cooking protocols, namely flour proportions, temperature, cooking, and fermentation time, can allow adjusting production variables and optimization to potentially overcome the decrease in sensory quality and preserve consumer acceptance.

## 1. Introduction

Bread is an essential food in the diet of many people around the globe [1,2,3], with results in average consumption of 59 to 70 kg per year per capita [4], which is due to the versatility of its preparation, relatively low cost, and the high energy supply [5]. Various pastries have been developed depending on the different baking procedures and ingredients used in each region or culture [6]; nevertheless, the key ingredients are flour, water, yeast, and salt [7].

**Figure 1:**
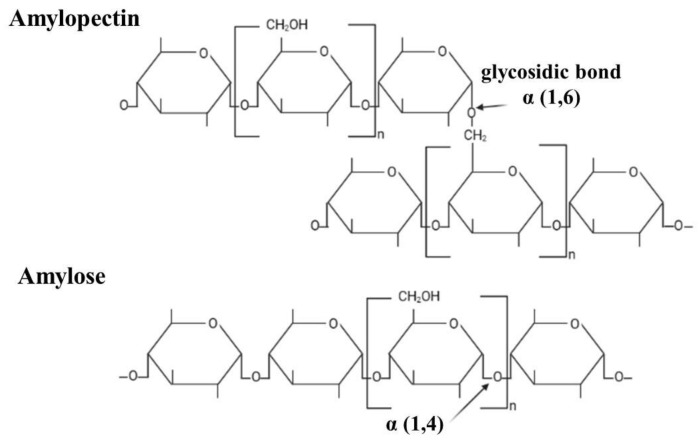
The chemical structure of the polymers presents in starch.

Bread is considered a solid, heterogeneous, and unstable foam, constituted by crust and crumbs, whose main components are starch granules displayed in a continuous proteic matrix [8]. These macromolecules comprise amylose and amylopectin (Figure 1) [9]. Amylose is a linear polymer formed by glycose monomers linked by α-1,4 glycosidic bonds, whereas amylopectin is a branched polymer with α-1,6 bonds [10]. Amylose concentrations in cereals range from 18 to 33%, while amylopectin concentrations range from 70 to 80% [11].

The type of flour and leavening agent in breadmaking play an essential role in determining its ultimate quality. Whole grain flour displays better overall nutritional quality due to its high fiber content, micronutrients, and bioactive compounds compared to refined flour [3]. The most common grain for breadmaking is wheat [7,12] since wheat flour creates a dough with desired rheological properties when mixed with yeast [13]. Furthermore, approximately 40% of the world’s population consumes wheat because of its essential nutrients [14]. Therefore, this grain represents 27% of global cereal production, given the adaptability of this crop to a wide variety of climatological conditions [15]. However, wheat contains gluten, which introduces limits on wheat consumption. Gluten is a protein that is difficult to digest and causes high intolerance in people with celiac disease [2]. As a result, there has been a push to develop bread with gluten-free grains such as rice or corn flour [16]. Another problem with bread is the high salt content in many products [17] and low fiber and essential amino acid levels, causing adverse health effects. In recent years, studies have addressed these issues by decreasing the amount of salt [18] and incorporating various fiber-reach flours from legumes such as lentils [19], white beans [20], soy [21], chickpeas [22], or dietary fibers. These approaches aim to increase its nutritional value and decrease the glycemic index caused by refined flour.

Nevertheless, altering the bread formula using gluten-free flour changes the features consumers accept, such as viscosity, elasticity, and volume [23]. In addition, changing the wheat flour shortens the shelf life of bread products [24]. For this reason, the food industry has a significant challenge in developing a product with high nutritional value that does not disturb the organoleptic characteristics of traditional high-quality bread. This work aims to survey bread’s rheological and nutritional aspects, the trends in bread making, and the modifications carried out by incorporating alternative flours such as the ones derived from gluten-free grains and legumes.

## 2. The Importance of Bread in the Global Diet

Wheat (*Triticum aestivum*) is a very important cereal in Europe; one of the main reasons is that it is the main producer with a total of 123 million tons, followed by China with 96 million tons and in third place India with 72 million a year [25].

**Figure 2:**
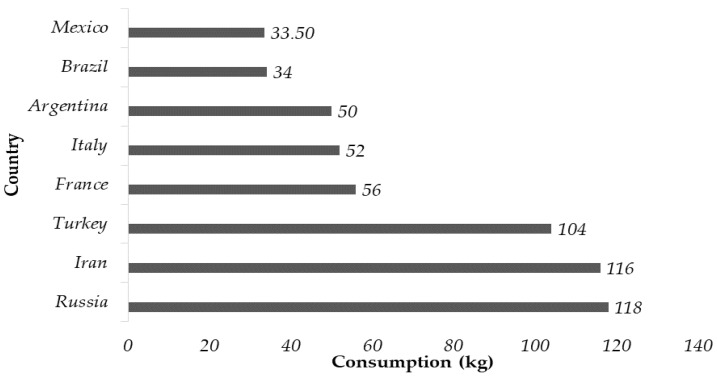
Different countries’ annual per capita bread consumption. Data reported in 2021 for Russia [29], 2019 for Iran [30], 2019 for Turkey [27], and 2019 for France [31]. Data reported in 2019 for Italy [25], 2022 for Argentina [32], 2020 for Brazil [33], and 2020 for Mexico [7].

Wheat is processed into flour to produce cookies, cakes, pasta, and bread [26]. Bread represents a staple of the global diet, with a consumption of over nine billion kilograms per year [17] In developed countries, the average consumption is 70 kg per capita per year [27]. One of the reasons for the high wheat consumption in the world is high production, making it an affordable cereal [26]. As shown in Figure 2, the largest consumers are Russia, Iran, and Turkey. The biggest consumers are Russia, Iran, and Turkey.

There is a wide variety of bakery products. For example, Mexico is one of the countries with the most types of bread in the world, including around 2000 types of sweet and 300 types of salty breads [7]. Mexican bread’s popularity is mainly due to its low cost [28].

Despite the increasing interest in whole grain bread due to its high fiber content, which is beneficial to health, white bread continues to be widely accepted worldwide, given its sensory attributes and affordable cost [28]. As shown in Figure 2, the current annual consumption of bread in Mexico is 33.5 kg per capita, of which about 70 to 75% is white bread, represented mainly by sweet bread and other pastries [7].

### 2.1. Current Nutritional Value of Bread

Whole wheat bread is rich in polysaccharides [34], proteins, phytochemicals, vitamins, and minerals, compounds that can positively affect health [15]. However, consumers might reject this kind of bread due to its taste. Another negative aspect is that whole wheat bread has low amounts of essential amino acids such as lysine and threonine [34]. Therefore, the research and baking industry is tasked with optimizing technological procedures in breadmaking to enhance its organoleptic characteristics, nutritional quality, and shelf life [35].

When the wheat is milled to obtain the flour, the fibrous layers of the grains are discarded, eliminating macro and micronutrients [36,37]. Whole wheat is formed by bran, germ, and endosperm [15]. Without the bran and germ, approximately 45% of the grain protein is lost, as well as 80% of fiber, 50–85% of vitamins, 20–80% of minerals, and up to 99.8% of phytochemicals [37], resulting in baked bread with deficient amounts of proteins, low-resistant starch, and dietetic fiber, as well as high concentration of carbohydrates and glycemic index [38]. Consequently, adding fibers, vitamins, and minerals to refined cereal products through enrichment and fortification procedures has become essential, offsetting losses by refining.

Nowadays, the fortification of the bread is necessary because evidence demonstrates that the intake of the removed milling discards, especially fiber, helps prevent certain cardiovascular diseases, cancer, and obesity [39]. Thus, food technology investigations have focused on improving the nutritional quality of wheat flour by adding protein-rich sources such as legume [19] or insect [40] flours.

### 2.2. Glycemic Index

Cereals represent an essential carbohydrate source in the human diet [41], providing over 50% of the daily calories worldwide [42]. The rapid body glycemic response of a determined food depends on factors such as the size of particles, cooking method, starch structure, and intrinsic biomolecule quantity (dietetic fiber, proteins, and fats). These biomolecules affect blood glucose levels postprandially, which leads to the identification of foods that substantially influence blood glucose levels and thus, to the definition of their glycemic index (GI) [41]. Jenkins et al. described GI as a classification method for foods with sugars that increase blood glucose levels, leading to hyperglycemia [43]. Foods are scored according to measures of glycemia increase; the ones over 70 are considered high GI, those ranging from 56 to 69 as medium GI, and those below 55 as low GI. These values were estimated using white bread as a standard food or glucose with GI of 100 [11]. After the intake of high-GI foods, a glucose peak in the blood is expected within the first two hours [42].

Wheat bread has an elevated content of fast-digestion polysaccharides, turning into a high-GI food [44]. High postprandial glycemia can be the cause of various chronic diseases, especially type 2 diabetes, which is frequent around the world [45]. Therefore, it is necessary to fortify bread with ingredients to reduce the GI and benefit health [44].

As a result, gluten-free products are more prominent in the market due to the increased diseases related to ingesting gluten and the change to a healthier lifestyle [1].

## 3. Biochemistry of Gluten Synthesis

Gluten is a protein only present in wheat, rye, and other baking-related products. This protein is the base of whey batter [46]. The total protein percentage in wheat flour ranges from 7 to 15%, of which about 80 to 90% is gluten [47]. The Codex Norms describes wheat gluten after hydration as a highly viscous protein [48].

Gluten is a protein complex composed of proline (10%), glycine (20%), and glutamine (35%, which is the most abundant amino acid) [47]. The rheological properties of wheat can be attributed mainly to the endosperm proteins, which make gluten [13]. Gluten is an alcohol-soluble fraction of the total proteins in grains, and it is named after the type of cereal, e.g., if it comes from wheat, those proteins are known as gliadins; in barley, it is named hordeins, whereas in oats, avenins [49].

Gluten proteins are often classified according to their solubility. Water-alcohol-soluble proteins are gliadins; the insoluble ones are glutenins [47,50]. Gliadins and glutenins are well known for their properties; gliadins provide viscosity to the dough, while glutenins provide stability and elasticity [47,51]. Gliadins comprise 30 to 40% of flour proteins [50].

## 4. Physicochemical and Rheological Properties of Bread Ingredients during Elaboration

Plenty of bread variants exist worldwide, and basic ingredients include grain flour, water, yeast, another leavening agent, and salt [8]. Wheat (*Triticum aestivum*) flour is among the most widely used flours in the bakery industry [7,52] due to its high baking efficiency compared to other grains [35] and high levels of micronutrients [26].

Wheat flour is the major raw material in bakeries [52] due to the aforementioned complex proteins formed by glutenins and gliadins that produce gluten when mixed with water [7]. The gluten formation brings out the needed rheological properties, creating a dough ready to produce bread after adding yeast [50]. Dough viscosity is due to gliadin, while dough elasticity is due to glutenin connections [53]. Because of these features, keeping these proteins in the bread formula is crucial for consumer acceptance [23].

Whole wheat flour makes dough and bread with different characteristics than those with refined flour, including less bread volume, more toughness of the crumb, thick texture, and darker and distinct taste and smell, resulting in bread with features hardly accepted by consumers [54].

On the other hand, fats constitute another important ingredient in breadmaking. Oils and fats are often added to the dough to help in processing and storage, representing up to 5% of the dough mixture [55].

Water is also essential for mixing raw materials to form a homogeneous, firm, and elastic dough. The amount of water depends on the chosen type of flour. The temperature of the water should be considered because yeast is only active in the temperature range of 0–55 °C; however, the most favorable temperature for fermentation is between 27–38 °C [56].

*Saccharomyces cerevisiae* is the preferred yeast for foods, beverages, and breadmaking [57]. In the bakery industry, yeast is a major leavening agent that enlarges volume [58,59,60] by producing carbon dioxide through the alcoholic fermentation of sugars and reducing the time needed to obtain the desired bread volume [60]. Besides improving dough’s rheological properties, yeast produces flavor initiator compounds [61].

Finally, another key factor in bread and yeast-related products is sodium chloride or salt, which provides a pleasant and expected taste to pastries [62], and improves dough consistency [63] by strengthening gluten and controlling fermentation [62]. The salt–gluten bond is crucial to obtain a good quality crumb [63].

### 4.1. Sensory Factors That Determine Bread Acceptance

Freshness is the first bread acceptance determining factor, encompassing adequate taste, smell, and texture [5]. The taste perception is related to the aroma during digestion [64].

Taste is the main attribute of accepting bread. During the fermentation and baking processes, volatile and non-volatile compounds, such as alcohols, esters, and heterocyclic compounds, are responsible for the taste [65].

Like the taste, the aroma is an important element that determines the final quality of the bread and its acceptance by consumers [66]. The scent has been carefully researched to identify volatile compounds, and close to 540 volatile compounds have been detected, mainly alcohols, aldehydes, esters, ketones, acids, pyrazines, and pyrroles [52]. In the breadcrumb, the aroma develops mainly with the fermentative activity of the yeast and the oxidation of the fats of the flour. In the crust of the bread, the aromatic compounds are formed due to the Maillard reaction in the baking process [67]. The amount of volatile compounds formed will depend on fermentation temperature parameters, fermentation time, and amount of yeast [68].

The texture is a crucial sensorial feature in breadmaking. It has been defined as a “group of physical characteristics that are outlined by touch, related to deformation, disintegration, and food fluidity after applied force, and it is measured objectively by force, time, and distance functions” [69].

The mechanical properties of crumbs determine the consumer perception of bread quality [70]. The crunchy texture is related to low humidity levels and water activity. When gluten and starch matrices are set in a vitreous state, they make the bread more prone to fractures [71].

#### 4.1.1. Kneading Technology

The elaboration of bread consists of three steps: kneading, fermentation, and baking [51]. Kneading the dough is a significant step in the process. Kneading the dough determines elasticity and viscosity parameters [72].

Dough results from mixing water and wheat flour. The rheological properties of the dough are determined by the applied mechanical force [51]. A fresh batter has minute air bubbles that expand when yeast releases CO_2_ during fermentation, increasing the bubbles’ size and stiffness and making them softer [73]. The temperature of the dough is an important factor because it determines the growth rate of the *S. cerevisiae* yeast. The optimal temperature is from 34 °C to 38 °C, with a pH of 4.0 to 5.2 [74].

#### 4.1.2. Fermentation

Fermentation comes from the Latin *fermentum,* which means “natural decomposing process”, where chemical changes occur because of certain microorganisms of vegetal or animal origin. Traditionally, this process is employed to conserve and transform food flavors, textures, and smells [75].

Various techniques and fermentation factors affect sensorial features and the final quality of products. The preferred leavening agents or substances that can incorporate air in baked products are active dry yeast, distillers’ yeast, sourdoughs, and chemical agents of fermentation [76].

*S*. *cerevisiae*, a microscopic organism that can be found in different presentations (fresh, instant, active, and liquid forms) [77], transforms fermentable carbohydrates present in batter, producing CO_2_ and ethanol, two products responsible for fermentation and enlargement of batter volume [59], aiming to upgrade the rheological properties and flavor of the forming bread.

**Figure 3:**
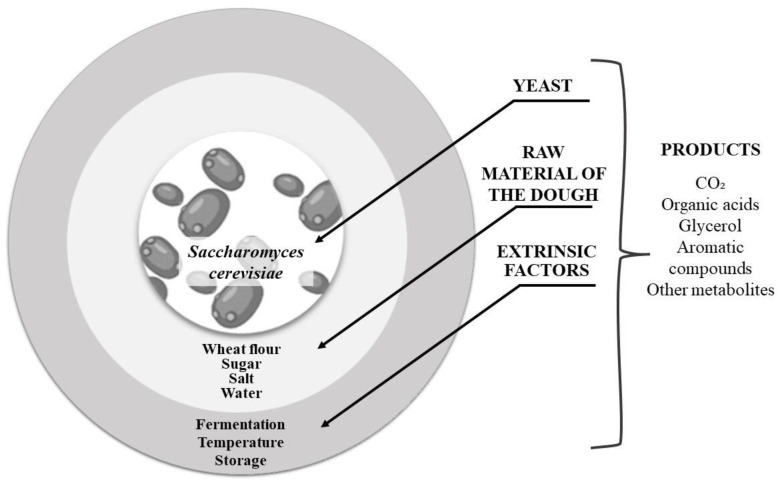
Dough components and factors that influence fermentation.

Sugars play an important role in causing dough fermentation. The dough is formed from raw materials and yeast (Figure 3). These biomolecules may be present in flour or externally added to the formula. In sweet bread, saccharose can be incorporated up to 30%, resulting in high osmotic pressure, which may be detrimental to yeast fermentation [59].

Yeast products, such as acetic acid, lactic acid, succinic acid, H_2_O_2_, and ethanol, tend to accumulate and cause the pH of dough to drop, inducing changes in the physical features of dough, such as reduction of extensibility in the case of acetic acid and succinic acid [78].

#### 4.1.3. Baking

Baking is the last step in breadmaking, where the raw dough will be modified through heat treatment, resulting in a pleasant product for consumers [79]. Baking is a traditional step where water, starch, proteins, and heat are the variables that determine the quality of bakery products [80]. Usually, ovens or heating machines are employed to raise temperatures to 260 °C for baking [81].

In this process, external variables can be measured and controlled to warrant the best bread quality, with temperature and humidity being the most important. These variables fluctuate along the day and change with the seasons, impacting the baking process [82].

A series of physicochemical and biochemical modifications occur during baking; starch undergoes a complex transformation known as gelatinization [35]. Starch gelatinization in cooking impacts starch susceptibility to enzymatic degradation [35].

Firstly, amylases are unfolded reversibly into an inactive state. After prolonged heat exposure, proteins denaturize irreversibly. The inactivation speed is correlated to the water content and bread heating speed [83].

High temperatures and low moisture in cereal-based products promote chemical reactions among the ingredients of bakery products, including Maillard reaction and caramelization [47,83]. These reactions cause desire and undesirable changes in the final product [84]. The desired changes include those that enhance sensory features, promoting the flavor, color, and smell of the final product, which are well appreciated by consumers [81].

#### 4.1.4. Crusting

The crust is the external layer of bread. This part is the hardest, driest, and darkest because of the baking temperature and time [85]. The crust determines the quality and shelf-life of products and is formed after evaporation during baking [86].

Crust and crumb come from the same dough, but the sensory features differ by the moisture percentage at each part [71]. When the dough is placed in the oven, water evaporates quickly in the external layer. Hence, the crust has a lower water proportion than the crumb [71]. The color and flavor of the crust are due to the Maillard reaction [85] after rapid dehydration and high-temperature exposure [84].

### 4.2. Browning Reactions

At temperatures higher than 50 °C, pH ranges from four to seven, and in medium humidity, the Maillard reaction is developed [87]. This reaction occurs in the presence of amino acids and reducing sugars under baking conditions [47,84]. It plays a significant role in the development of compounds responsible for the aroma and color of the product [88], but also in forming harmful compounds [71], e.g., acrylamide [47], which is a potential human carcinogen [84].

**Figure 4:**
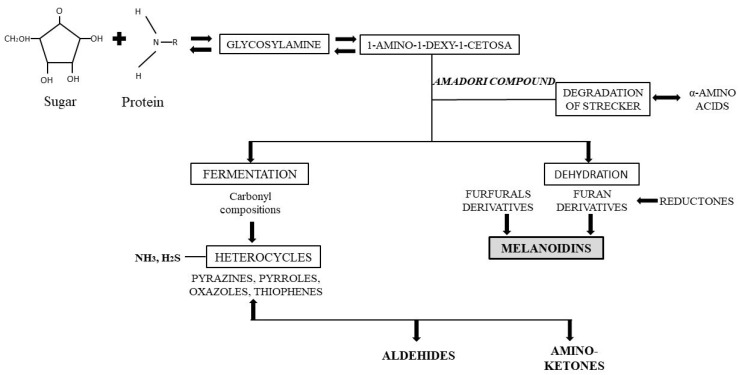
Formation of flavor compounds during the Maillard reaction.

A protein reacts with sugar during browning reactions, creating glycosylamine (Figure 4). This chemical compound loses a water molecule to form a Schiff base, which undergoes an Amadori transposition reaction. Amadori’s compounds decompose into a reorganized sugar and an amino acid residue [88]. At the end of the Maillard reaction, reagents and part of the sugars condense to form brownish nitrogen compounds [89] with high molecular weights [90]. These molecules are responsible for the brown aspect of baked products [47]. The different molecules generated in the degradation of the Strecker as the final stage of the Maillard Reaction are a group of volatile heterocyclic compounds such as: pyrazines, pyrroles, pyridines, among others, and these contribute to the taste and aroma of many foods [91]. Pyrazines and pyrroles contribute directly to the aroma of bread [92]. Pyrazines are responsible for the nutty taste and roasted aroma [93].

The Maillard reaction not only improves the sensory quality of bread, but it has also been shown to have antioxidant benefits and antimicrobial properties [94]. Melanoidins are high-molecular-weight molecules and contain nitrogen. This molecule is obtained in the final phase of the Maillard reaction. Melanoidins not only improves the taste, aroma, color and texture of bread, several studies suggest that melanoidins have antioxidant, antimicrobial properties [90,94,95], and presents prebiotic and antihypertensive activity [96].

Another reaction during baking is caramelization, which involves chemical changes that combine complex molecules that determine the color (melanoidin) and smells [97]. Caramelization happens after sugar degradation at 120 °C [87].

### 4.3. Starch Gelatinization and Retrogradation

Starch undergoes structural modifications related to gelatinization and retrogradation [98]. Starch is a highly organized structure with high stability because of the interaction of the constituent water-insoluble polysaccharides. However, when the grain is exposed to heat, the liquids are absorbed in the amorphous inter micellar zone of the amylopectin (less organized regions since hydrogen bonds are not as stable and abundant), triggering a volume enlargement that can be observed via microscope. Depending on the type of starch or when the reaction reaches 65 °C, the granule hits its maximum volume and loses orientation (the X-ray diffraction pattern disappears), resulting in gelatinization [99].

During bread baking, a fraction of the starch can gelatinize, but it only gelatinizes completely with high percentages of water (up to 65%) and a temperature around 70 °C. If not, enough water is incorporated into the batter mix, the reorganization of starch molecules can only occur at higher temperatures (up to 100 °C) [28].

Consolidating bread or starch-containing foods triggers a reorganization of this polysaccharide, denoted as retrogradation [28].

**Figure 5:**
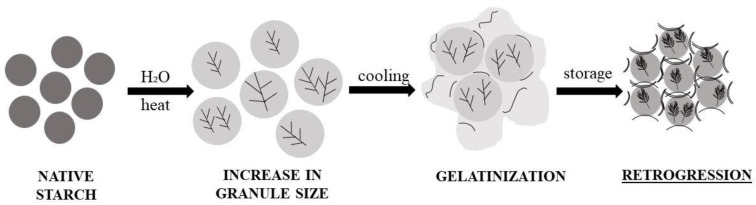
Starch gelatinization and retrogradation.

As shown in Figure 5, starch retrogradation is a reordering molecule process that determines bread firmness or hardening [8]. This event happens after starch hydration with warm water and chilling, so the amylose and amylopectin are reincorporated into a molecule differently [100].

The use of emulsifiers might be used in bread cooking as additives to assist dough strengthening and crumb softening by interacting with the starch. Some emulsifiers used in bread formula are diacetyl tartaric acid ester of monoglycerides (DATEM), sodium stearoyl lactate (SSL), polysorbates, and mono- and diglycerides [54].

Another way to prevent the rejuvenation of bread is the use of enzymes; they are used in gluten-free doughs to improve the quality of bread [101]. For example, α-amylases have been used to improve the fermentation process, increase the volume of bread, and decrease the hardening of bread [102].

## 5. Bread Enrichment/Enhancement of Nutritional Value

Currently, consumers look for products that cover their daily necessities. For this reason, several studies have targeted the enhancement of the nutritional value of bread [2,103,104]. Adding cereals and seeds with high protein and mineral value or modifying methods to upgrade cereals’ fermentation or germination helps digestion and increases minerals’ bio-disponibility [105]. The use of vegetable sources to increase the protein value of bread is due to its low cost compared to proteins of animal origin [106]. As shown in Table 1, incorporating faba bean, chickpea, soy, and amaranth flour in a wheat bread formulation significantly increased the protein, mineral, and fiber values concerning the control bread. In addition, the formulations in which the highest percentages of protein were obtained were bean and chickpea flour, with an increase of 6.8 and 6.4%, respectively.

### 5.1. The Use of Alternative Flours

Traditional bread lacks essential amino acids such as threonine, tryptophan, and lysine [105]. In addition, making bread from refined wheat flour or without wheat bran reduces fiber and mineral amounts and therefore also reduces its nutritional value [34]. Many studies have proposed increasing bread protein value by adding protein isolates from different sources to constitute up to 80% of the protein content [16].

Another attempt to improve bread’s nutritional value is partially substituting wheat flour with other grain or cereal flour that contains needed amino acids, reducing protein malnutrition [109].

Legumes play an important role in diets worldwide, given their protein content, which oscillates from 7 to 40%, against cereals’ protein content of 7 to 13% [110]. Soy is one of the legumes most often chosen in pastries because of its high quantities of proteins, fats, and other constituents that help diminish the risks of coronary diseases and different types of cancer [109]. The protein content of soy flour is 35 to 45%, which is needed for a well-functioning human body [21]. Studies have revealed that adding soy flour enhances bread’s nutritional value and quality [4,107,111].

Another flour used in baked products is chickpea flour (*Cicer arientum*) [112], a legume that sits 5th in the world for its elevated nutritional value and low cost. Chickpea flour is rich in proteins and carbohydrates, 22 and 50%, respectively [106], and contains oligo-elements such as calcium, potassium, iron, and magnesium [113], all essential minerals for metabolism in living beings [114]. Chickpea flour is composed of amino acids that wheat flour lacks, such as leucine; therefore, it is an ideal alternative to supplement the protein value of wheat flour [113].

Similarly, lentils have been incorporated into baked products mainly because they provide little flavor [115] and have a high protein value, including essential amino acids such as lysine, leucine, arginine, glutamic, and aspartic acid [19], with even higher percentages of these (30 to 33%) during germination [116]. Additionally, lentils have great benefits because they are a source of β-glycans that help to reduce GI and maintain body weight [34].

Finally, studies have reported the use of bean varieties in baked products [117] because of the presence of essential amino acids and sources of minerals such as iron [20], which is an essential component for hemoglobin to transport oxygen in the blood [116].

### 5.2. Dietetic Fiber

Dietetic fiber (DF) is acknowledged as a crucial vegetal component in the human diet [79]. This carbohydrate polymer is often present in vegetal cell walls, differing from simple sugars like mono and disaccharides [118]. Dietary fibers are represented by hemicellulose, cellulose, resistant starch, inulin, pectin, and gum [79].

Cereals are an important source of DF. Hence, pastries like whole grain or not-refined flour bread have high DF. Nonetheless, consumers are less accepting of these products given their modified sensorial characteristics, principally the flavor, and because the bread is less voluminous [118]. In wheat, DF is found mainly in the external layer or pericarp, i.e., bran, representing over 15% of the total grain weight [119]. There are two types of food fiber: soluble and insoluble [26]. The last contains significant amounts of cellulose (around 32%), hemicellulose (around 30%), and lignin (around 16%) [119].

Dietetic fibers must be included in bread batter to increase shelf life. This is due to the ability of fiber to retain water, delaying fat decay [79]. According to the WHO, a person must have a minimum daily intake of 25 g [120] because of its beneficial impacts on health. Fiber also has a prebiotic effect, stimulating and supporting microbiota growth [121]. Fiber consumption is deficient in many countries. Therefore, several studies added wheat bran as a source of dietary fiber in various food products, including baked products [119].

Navrotskyi and collaborators evaluated the impact of the chemical and rheological properties of bread with the addition of wheat bran as a source of insoluble dietary fiber, obtaining favorable results in the amount of fiber, proteins, and minerals, but unfavorable results due to the decrease in volume affecting the texture [122]. Similarly, Liu and collaborators analyzed the rheology of wheat flour dough by incorporating wheat bran at different percentages (0, 3, 6, 9, and 12), proving a reduction in viscosity and increased water absorption with increasing wheat bran percentage [123].

Oatmeal is another cereal used to increase the amount of dietary fiber in bread [124]. This cereal, rich in lysine, threonine, tyrosine, and tryptophan proteins, also contains β-glucans, a soluble dietary fiber [125]. Chauhan et al. (2018) evaluated the addition of oat flour in different percentages (0, 10, 15, 20, and 25) in preparing wheat bread. The study compared the different bread formulations’ organoleptic and rheological characteristics, as well as nutritional compositions. This addition will result in a better sensory acceptance of wheat bread containing 20% oatmeal, simultaneously decreasing the amounts of carbohydrates and improving the quality of proteins, fiber, fat, and ashes. However, the bread volume was affected by increasing the percentage of oatmeal [126].

Studies have incorporated legume flour into baked goods to increase the dietary fiber content [34,127]. Chickpea flour, an important dietary fiber source, was incorporated at three levels (7.5%, 15%, and 30%) in a wheat flour sandwich bread. Consumers accepted all the formulations as they did not present negative sensory changes. The formulation with the highest percentage of added chickpea flour gave a better nutritional value, especially in dietary fiber [128].

### 5.3. Resistant Starch

Starch is significant in human nutrition since it provides 25% of the calories needed. Catalytic proteins break starch bonds, obtaining glucose. However, certain starches cannot be unfolded; thus, the enzymes cannot break them into smaller glucose units, and they reach the colon, where microbiota ferment them, producing short chains of fatty acids with prebiotic effects. This starch fraction resistant to the digestive process is called resistant starch (RS) [129,130].

According to Roman and Martínez (2019), RS is a starch fraction that resists the digestive process. RS remains because enzymes cannot break it into smaller glucose units; thus, it reaches the colon intact, entirely fermented [130].

From the point of view of their origins, RS can be clustered into five types: type 1 (RS 1), physically inaccessible starch, found in vegetal structures such as grains and seed partially milled; type 2 (RS2), native starch granules, present in raw potatoes and bananas; type 3 (RS 3), retrograde starch found in potatoes and bread; type 4 (RS 4), chemically modifies starch of unnatural origin and obtains for esterification or crossover, and type 5 (RS), amylose-lipid complexes [131].

Resistant starch has proven to have health benefits related to DF [121]. Therefore, there is a great interest in using RS for bakery products as a functional ingredient, provided that it minimally changes the organoleptic profile since it has no flavor and is white [132].

Some benefits of RS observed in the gastrointestinal tract include increased feces volume, which contributes to a proper evacuation rate, avoidance of constipation, and improved intestinal microbiota functioning, which results in better immune system activity [133]. RS can be chosen as a low-calorie alternative diet component to increase fiber content and thus diminish food intake caused by the prolonged satiety feeling [134].

Several studies have been published on bread with added RS. Barros et al. (2018) used corn flour (which contains 60% RS) incorporated at 10, 15, and 20% of wheat bread, and demonstrated that the formulation with 15% did not negatively impact the quality of the bread, presenting rheological characteristics similar to the control bread [132]. In another study where resistant corn starch type II with different percentages (10, 20, 30) was integrated into the bread, crumb moisture loss, specific volume, and porosity were evaluated during storage. The bread with percentages of 10 and 20 obtained good volume quality, porosity, and slower moisture loss; on the contrary, the bread with 30% RS showed a high water loss and firm crumb, resulting in poor-quality bread [135].

### 5.4. Salt Reduction

Common salt, or sodium chloride, NaCl, is a chemical compound widely used in the preparation and conservation of food for its water-absorbing and antibacterial properties [136].

Sodium in the human body assists in nervous impulses and controls blood pressure and volume [137]. Every 2.54 g of salt provides 1 g of sodium [136]. Being present in most foods consumed daily, salt can easily cause an excessive sodium intake, which might have detrimental consequences on health, causing high blood pressure and various cardiovascular diseases [17,26], like stroke and kidney problems [18]. Excess sodium can also damage bone tissue by expelling calcium, increasing the possibility of bone fractures [138].

The World Health Organization recommends a daily salt intake of no more than 5 g, but most people consume more than 10 g a day [139]. Therefore, it remains mandatory to diminish the consumption of foods with high salt content [137].

Bread is an essential food consumed daily [136]. For each 100 g of bread, there may be up to 2 g of salt or 400–800 mg of sodium [17] Since bread is a popular diet component, it becomes urgent to diminish the added salt levels to prevent the health issues mentioned above [140] because bread can provide almost 30% of the daily salt intake [17]. Unfortunately, adding lower amounts of salt to the bread formulation affects its sensory characteristics, such as the color of the crust, smell, and consistency of the crumb [18]. Also, salt plays a fermentation-controlling function; therefore, the food industry has a big challenge ahead to lower salt percentages with no disturbances in the quality of products [136]. It has been shown that by decreasing the amount of salt in baked goods, organoleptic characteristics are affected, reducing the acceptability of these products [18,141].

Studies have proven that adding mother dough to the fermentation process diminishes 1.5% of the salt amounts in bread with no alterations in sensorial attributes or other quality measures [26]. Reißner et al. (2019) [142] partially used other minerals (KCl, CaCl_2_, and MgCl_2_) to substitute the salt, demonstrating that using KCl with 50% does not show differences in bread volume, dough stiffness, and crumb quality, comparable to control bread. On the contrary, using CaCl_2_ in lower percentages causes undesirable flavors [142].

Another study with the partial substitution of NaCl with KCl obtained negative results of the flavor attribute when using percentages above 40% of KCl, recommending only KCl at 30% to prevent sensory changes in the bread [143].

### 5.5. Changes in Organoleptic Characteristics

Developing beaked products with legume flour helps increase amino acids but carries disadvantages in technological processes, such as the extension of mixing, baking, and fermentation times. Another adverse point is the consumer acceptance grade that diminishes after changes in the final product’s texture, color, and flavor [144]. Studies have demonstrated the importance of including wheat flour in bread formulas because gluten is important for fermentation, rheological properties, and better quality [145] Therefore, adding the alternative flours in low percentages would avoid drastic changes in rheological and sensory properties [146]. Table 2 summarizes wheat bread’s organoleptic changes and protein values upon legume flour incorporation. The percentages of protein vary from 10 to 19%; the highest values are reported for fava beans (*Vicia faba* L.) with 19.6% and amaranth flour with 18.1%; this shows that legumes are richer in protein than cereals [107].

### 5.6. Shelf-Life

Bread freshness decreases rapidly, making bread a product with a short shelf life due to physicochemical and microbial changes [5] The main technological changes during aging [149] are caused mostly by migration, retrogradation of amylopectin, and gluten-starch interactions [132]. All these alterations cause a decrease in crust crispiness, an increase in crumb hardness, an overall reduction of organoleptic properties [149], and a loss in the capacity to retain water [132]. Besides starch retrogradation, other events happen, such as water migration from the inside to the bread’s exterior and interactions among bread components that conduct protein changes and produce a starch-protein matrix [8]. These technological modifications occur during bread storage, possibly diminishing bread quality [70].

Bread has an average shelf life of 4 to 10 days since most of these baked products present a high water activity (aw 0.95). For this reason, the industry faces the challenge of increasing shelf life [150].

Some innovative processes that have been used to improve the physicochemical properties and increase the shelf life of bread are the incorporation of specific enzymes resulting in an improvement in bread’s texture and shelf life, delaying hardening and maintaining moisture, and allowing prolonged freshness [151]. Similarly, it has been reported the use of gases such as carbon dioxide, nitrogen, and oxygen to modify the atmosphere inside the bread packaging, which can slow down the deterioration of the product by reducing the rate of oxidation and microbial growth [152], or active packaging, developments with improved barrier properties helping to keep the freshness of the bread for longer by protecting it from air, light, and moisture more effectively than traditional packaging [153]. Also, packaging film with antimicrobial solutions helps prevent mold growth, thus prolonging the shelf life [154,155].

## 6. Conclusions and Future Perspective

Because of the high consumption of baked products, especially bread, it is necessary to raise awareness among consumers to choose non-refined flour products that are richer in fiber and lower in salt. Consumers should be encouraged to consume healthier products that have an added value aiming to prevent harmful illnesses. However, by incorporating alternative raw materials, some changes in color, flavor, or texture may become evident and, thus, be rejected by consumers. Consequently, the food industry, along with the development of new product areas, has the challenge of satisfying the necessities and demands of users. Thus, it has become mandatory to perform changes in the technological procedures or reformulate with additives and enzymes to allow the addition of higher percentages of other types of flour richer in dietetic fibers and proteins and therefore obtain a food product with human health benefits.

Beneficial products for consumers rely on adding additives and enzymes, increasing the percentage of flours rich in dietetic fibers and proteins. Thus, there is a need to change the technological procedures and reformulate the existing products.

The great challenge is to change the taste preferences of consumers towards healthier options, in the same way that incorporating high-quality ingredients bread can be more expensive, which can affect its accessibility.

Given the challenges, it is proposed to continue reformulating these products of high consumption with desirable organoleptic effects and favorable results for preventing cardiovascular diseases caused by excess salt and decreasing glycemic index. However, consumer acceptance is a weakness in the current knowledge on new bread product development, including up to 50% of other sources as raw materials. Therefore, optimizing this foodstuff will require deep studies beyond sensory evaluation and consumer acceptance, like gastrointestinal digestion and accessibility assessments and bioactive and prebiotic effects after intake.

## Figures and Tables

**Table 1:** Effect of including whole vegetable flours on the nutritional value of a control wheat bread.

	Formulations Wheat Breads							
Parameter	Control	15% Faba Bean Flour	Control	20% Chickpea Flour	Control	15% Soy Flour	Control	25% Amaranth Flour
Moisture (%)	32.20	26.55	12.70	11.40	27.90	26.51	27.80	28.60
Protein (%)	12.89	19.69	8.90	15.30	9.80	12.9	16.50	18.10
Fat (%)	1.06	1.23	1.80	2.90	3.30	4.1	0.07	0.70
Ash (%)	0.96	2.55	0.67	2.11	1.70	2.2	1.70	3.00
Fiber (%)	0.43	1.00	0.60	4.70	0.29	0.38	3.90	6.30
Carbohydrate (%)	52.46	48.98	------	------	58.30	52.3	------	------
Reference	[107]		[106]		[23]		[108]	

**Table 2:** Effect of adding different flour types in organoleptic properties of wheat bread.

Flour Mixed with Wheat	Amount Added (%)	Organoleptic Changes	Water Absorption (%)	Protein (%)	Reference
Soy protein	15	Increment in hardness. Specific volume reduction.	61.60	10.20	[145]
Lentil (*Lens Culinaris Medik)*	20	Irregular and thick crumb. Decrease in dough stability and bread volume.	66.30	16.35	[114]
White bean (*Phaseolus vulgaris* L.)	20	Volume reduction. Increase moisture.	56.60	12.80	[20]
Faba beans (*Vicia faba* L.)	15	Increment in water absorption. Volume reduction Bread heaviness	67.30	19.69	[107]
Chickpea (*Cicer arietinum* L.)	15	Decreased dough stability. Decreased crumb texture. Reduced taste acceptance and color.	59.80	-------	[147]
White quinoa	30	Reduced porosity. Darker. Crumb hardness increment.	59.43	12.10	[148]
Amaranth flour	25	Volume reduction. Increase in firmness. Darker crumb.	60.50	18.10	[108]
Chickpea (*Cicer arietinum* L.)	30	More water absorption in the dough. Sticky and hard-to-handle dough. Volume reduction. Firmness in crumb. Darker crust and crumbs.	62.50	------	[22]

## Data Availability

No new data were created or analyzed in this study. Data sharing is not applicable to this article.
